# Fitness costs associated with infections of secondary endosymbionts in the cassava whitefly species *Bemisia tabaci*

**DOI:** 10.1007/s10340-017-0910-8

**Published:** 2017-08-22

**Authors:** Saptarshi Ghosh, Sophie Bouvaine, Simon C. W. Richardson, Murad Ghanim, M. N. Maruthi

**Affiliations:** 1 Natural Resources Institute, University of Greenwich, Central Avenue, Chatham Maritime, Kent, ME4 4TB UK; 2 Faculty of Engineering and Science, University of Greenwich, Medway Campus, Central Avenue, Chatham Maritime, Kent, ME4 4TB UK; 3 Volcani Center, ARO, HaMaccabim Road 68, PO Box 15159, 7528809 Rishon Le Tsiyon, Israel

**Keywords:** Cassava, Whitefly, *Arsenophonus*, *Rickettsia*, Fitness costs, Virus retention

## Abstract

We investigated the dual effects of bacterial infections and diseased cassava plants on the fitness and biology of the *Bemisia tabaci* infesting cassava in Africa. Isofemale *B. tabaci* colonies of sub-Saharan Africa 1-subgroup 3 (SSA1-SG3), infected with two secondary endosymbiotic bacteria *Arsenophonus* and *Rickettsia* (AR+) and those free of AR infections (AR−), were compared for fitness parameters on healthy and *East African cassava mosaic virus*-Uganda variant (EACMV-UG)-infected cassava plants. The whitefly fecundity and nymph development was not affected by bacterial infections or the infection of cassava by the virus. However, emergence of adults from nymphs was 50 and 17% higher by AR− on healthy and virus-infected plants, respectively, than AR+ flies. Development time of adults also was 10 days longer in AR+ than AR−. The whiteflies were further compared for acquisition and retention of EACMV-UG. Higher proportion of AR− acquired (91.8%) and retained (87.6%) the virus than AR+ (71.8, 61.2%, respectively). Similarly, the AR− flies retained higher quantities of virus (~ninefold more) than AR+. These results indicated that bacteria-free whiteflies were superior and better transmitters of EACMV-UG, as they had higher adult emergence, quicker life cycle and better virus retention abilities than those infected with bacteria.

**Electronic supplementary material:**

The online version of this article (doi:10.1007/s10340-017-0910-8) contains supplementary material, which is available to authorized users.

## Key message

- Cassava is a staple crop of millions of poor in SSA and its production is constrained by two whitefly-borne virus epidemics.
- Unusually high populations of whitefly are driving these epidemics.
- High numbers (~40%) of these populations were free of secondary bacterial symbionts.
- Here, we provide experimental evidence that bacterial symbionts negatively impact the biology of whiteflies by reducing adult emergence and delayed development.
- Whitefly population free of symbionts also retained higher titres of cassava mosaic virus.

## Introduction

Endosymbiotic relationships between insects and microorganisms can be unequal, in which one associate usually takes more than the other (Bourtzis and Miller 2003); however, in many cases these relationships remain uncharacterised. Members of the cryptic species of the whitefly *Bemisia tabaci* harbour many endosymbiotic bacteria including an obligate primary symbiont (P-symbiont), *Portiera aleyrodidarum* (γ-proteobacteria) and seven other facultative secondary symbionts (S-symbiont); *Arsenophonus*, *Hamiltonella* (γ-proteobacteria), *Cardinium* (bacteriodetes), *Fritschea* (Chlamydiae), *Rickettsia*, *Wolbachia* and *Hemipteriphilus* (α-proteobacteria; Chiel et al. 2007; Bing et al. 2013a). The prevalence of the S-symbionts vary depending upon host population, geographical location and host plants (Chiel et al. 2007; Gueguen et al. 2010; Pan et al. 2012; Bing et al. 2013b; Ghosh et al. 2015).

The interactions between the S-symbionts and *B. tabaci* are highly specific and varied with the symbiont, host population and geographical location. Infection by *Rickettsia* in the *B. tabaci* species, Middle East-Asia Minor 1 (MEAM1) in the USA, for example, provided fitness benefits with higher adult progenies, female bias and faster development time (Himler et al. 2011). However, a similar infection in Israel showed that infection with this bacterium increased susceptibility to insecticides (Kontsedalov et al. 2008) and provided higher heat tolerance (Brumin et al. 2011). Similarly, another symbiont *Cardinium* is detrimental to the MED species in China (Fang et al. 2014). The nature of interaction between the cassava whiteflies and its symbionts was yet unknown but was essential to understand the dynamics of cassava whitefly populations in sub-Saharan Africa (SSA).

Several *B. tabaci* populations infesting cassava in SSA spread viruses that cause the two most important diseases of cassava in Africa: cassava mosaic disease (CMD) and cassava brown streak disease (CBSD) (Legg et al. 2015). Epidemics of CMD and CBSD have been reported in the last 30 years which have affected the food security of millions of poor in SSA (Legg et al. 2015). Presence of endosymbionts such as *Hamiltonella* and *Rickettsia* can alter vector capabilities of *B. tabaci* by increasing virus acquisition, retention as well as transmission possibly due to the effect of GroEL protein (Gottlieb et al. 2010; Kliot et al. 2014). However, the role of bacterial symbionts in the transmission of viruses by the cassava whitefly has not been studied.

The rapid spread of CMD and CBSD epidemics in eastern Africa is, however, well documented and is believed to be driven by the unusually high numbers of whiteflies found on cassava plants. Recent surveys reported more than 1000 adults per cassava plant in many areas of central Uganda and around the Lake Victoria region of northern Tanzania (Colvin et al. 2004; Legg et al. 2006). A specific whitefly population called the sub-Saharan Africa 1-subgroup 1 (SSA1-SG1) was the predominant population in the epidemic regions (Legg et al. 2011, 2013). Interestingly, a high percentage (38%) of SSA1-SG1 were free of S-endosymbiont bacterial infections (Ghosh et al. 2015; Tajebe et al. 2015b). In contrast, another cassava whitefly population SSA1-SG3, found predominantly in coastal East Africa, has been less abundant (Jeremiah et al. 2015; Tajebe et al. 2015a) with an average of 10–20 whitefly adults per plant. Our recent results on the prevalence of S-endosymbionts showed a small proportion of SSA1-SG3 free of S-symbiont bacteria (13%) while the majority (87%) were infected with single or multiple infections (Ghosh et al. 2015).

We therefore set out to investigate the effects of the two most predominant S-endosymbiotic bacterial infections *Arsenophonus* and *Rickettsia* on the biology and the vectoring abilities of the cassava whitefly SSA1-SG3. Their effect on the expression of some whitefly immunity genes and on fitness costs was also investigated. The localisation of bacteria in the bodies of African cassava whiteflies was detected for the first time using fluorescent in situ hybridisation (FISH) technique. An understanding of the role of bacterial infections is essential to better understand cassava whitefly population dynamics in SSA and the viral diseases they spread.

## Materials and methods

### Whitefly colonies and cassava plants

Isofemale lines of *B. tabaci* infesting cassava were generated by random isolation of individual male and female with specific bacterial infections, and allowing sibling mating for developing into colonies. Whiteflies used in this study for generating isofemale lines have been maintained and inbreeding in cassava plants in the NRI insectary for 20 years (Maruthi et al. 2001). Genetic background of whiteflies was determined by sequencing mtCOI marker, and the presence of symbionts confirmed by PCR detection (Ghosh et al. 2015). Using these methods, two isofemale lines of SSA1-SG3 with identical genetic background, but differing in S-endosymbiont infection were developed. One colony was fixed (100%) with dual infections with the A3 strain of *Arsenophonus* and the R2 strain of *Rickettsia* (AR+) (Ghosh et al. 2015), and the other was free of S-symbionts (AR−). Both the populations were confirmed to be free of all other known symbionts infecting *B. tabaci* by PCR tests (Ghosh et al. 2015). The isofemale lines were maintained in insect-proof cages in a NRI quarantine insectary in the UK at 27 ± 3 °C, 60% relative humidity and photoperiod of L12:D12.

Cassava plants of the var. Ebwanateraka infected with the begomovirus *East African cassava mosaic virus*-Uganda (EACMV-UG) (family Geminiviridae) were grown in the NRI quarantine glass house. Plants were grown in 10 × 10 cm plastic pots containing John Innes no. 2 compost and soil in equal mixture at 28 ± 5 °C and 50–60% relative humidity. Two-month-old plants were used in experiments to compare fitness parameters (see below) of the two whitefly populations.

### Whitefly fitness assays

Two-day-old adult whiteflies were collected from SSA1-SG3 AR+ and AR− colonies using an aspirator and anesthetised using CO_2_ for 5 s. Insects from each colony were sexed separately under a stereobinocular microscope, and one female and two males from the same colony were transferred to 25-mm-diameter clip cages. The cages were then attached to a leaf of 2-month-old cassava plant var. Ebwanateraka for oviposition. The experiment was set up on 10 EACMV-UG-infected and healthy plants each for AR+ and AR− whiteflies for measuring whitefly fecundity and biology, and the experiment was repeated twice. The clip cages were removed after 12 days, and the whitefly eggs and nymphs were counted using a stereobinocular microscope. Only clip cages that had live females after 12 days were used for the analysis of fitness assays. Fecundity was measured on the 12th day immediately after removing clip cages.

The leaves with eggs and nymphs were enclosed in perforated bread bags to prevent contamination from other insects, and the number of nymphs developed and adults emerged was recorded every 3 days. The average number of eggs hatching was estimated based on the total number of nymphs emerged. The mean adult emergence (total number of adults emerged/total number of nymphs developed) and mean adult development time (from the first to final adult emergence) was also recorded for both whitefly colonies.

### Statistical analysis

All statistical analyses were done using the R software (R Development Core Team 2011). Mean fecundity (number of eggs laid) of the adult whiteflies and the proportion of nymphs developed were analysed using a generalised linear model with negative binomial errors and quasibinomial errors with logit link function, respectively. The mean proportion of adult emergence was also analysed using a generalised linear model with binomial errors with logit link function. The differences in means of adult emergence were compared by Tukey’s HSD test using the glht function from the multcomp package of R (Hothorn et al. 2008). The mean adult development time was analysed using a simple linear model. The mean differences in adult development time were compared by Tukey’s HSD test.

### Virus acquisition and retention

Adult SSA1-SG3 AR+ and AR− whiteflies were given acquisition access period (AAP) of 48 h on a 3-month-old EACMV-UG-infected plant var. Ebwanateraka in a large Perspex cage (50 × 50 × 100 cm). Twenty-five AR+ or AR− potentially viruliferous whiteflies were then transferred on to 2-month-old healthy cassava plants var. Ebwanateraka for 48-h virus inoculation access period (IAP). The experiment had three replications, and a total of 30 healthy plants were inoculated using each whitefly colony. Potentially viruliferous whiteflies were collected from all the experimental plants (after 48-h AAP and IAP, Maruthi et al. 2002) for virus detection and quantification in single whiteflies.

Total DNA from each whitefly female was extracted separately using 20% Chelex as previously described (Ghosh et al. 2015). Quantitative real-time PCR (qPCR) was performed with CFX96 real-time PCR detection system (Bio-Rad) with EXPRESS qPCR Supermix (ThermoFisher Scientific, UK). The whitefly α-tubulin gene (KC161212) was used as the reference for relative quantification. Multiplex detection of EACMV-UG and the whitefly α-tubulin gene in a single reaction with the hydrolysis probes (Table S1) was carried out to determine the quantities of virus retained in individual whiteflies. The assay was standardised using primers and probes in different concentrations and by comparing to singleplex reactions. The optimised multiplex assay consisted of 10 µl of 1X qPCR super mix, 300 nM of tubulin primers, 500 nM of EACMV primers, 100 nM of each probe and 3 µl of DNA extract from individual whiteflies in a 20 µl reaction mixture. PCR conditions of 95 °C for 2 min followed by 40 cycles of 94 °C for 15 s, annealing at 54 °C for 20 s and extension at 60 °C for 30 s were used for the assay. Each sample was tested in duplicates. The Cq values were determined by single threshold method, and relative virus quantities were calculated using the 2^−ΔΔCt^ method (Livak and Schmittgen 2001). A common SSA1-SG3 AR+ sample was used across the plates for calibrating virus quantities.

### Determining virus transmission efficiencies

Virus-inoculated plants were kept in cages for 60 days post-inoculation for the expression of symptoms. Leaf samples were then collected from virus-inoculated plants for testing for virus infection by qPCR. Total nucleic acids were extracted from leaves using the CTAB extraction method (Maruthi et al. 2002). Two µl of the DNA lysate was used for detecting EACMV-UG using the previously described primer pairs (see Table S1 in Supporting Information) and PCR conditions (Otti et al. 2016).

The mean quantities of EACMV-UG in AR+ and AR− whiteflies were analysed using a simple linear model with log_e_-transformed data to fit parametric analysis. Statistical inference was based on the resulting one-way analysis of variance (ANOVA). Statistical significance of the transmission efficiency and frequency of AR+ and AR− whiteflies carrying EACMV-UG after 48 h each of AAP and IAP was estimated in contingency tables using Fisher’s exact test at *P* < 0.05. All statistical analyses were done using the R software (R Development Core Team 2011).

### Relative quantification of whitefly immune genes by qPCR

Total RNA was extracted from pooled samples of 15 individuals (10 females and 5 males) in twenty replicates using TRIzol^®^ reagent (Pakkianathan et al. 2015) for quantifying immune genes in bacteria-infected and bacteria-free whiteflies. The extracted samples were treated with DNase I to remove genomic DNA according to manufacturer’s instructions. RNA yields were quantified using NanoDrop 2000. A total of 150 ng of whitefly RNA was used as template for first-strand cDNA synthesis using RevertAid H Minus First Strand cDNA Synthesis kit. Whitefly immune response genes (Table S2 in Supporting Information) were amplified using 1 µl of cDNA in qPCR using DyNAmo Flash SYBR green qPCR kit. All equipment and reagents used in this experiment were obtained from ThermoFisher Scientific Ltd., UK. Whitefly α-tubulin gene was used as the reference gene, and relative expression levels were calculated using the 2^−ΔΔCt^ method (Livak and Schmittgen 2001). Mean relative expression of immune response genes in AR+ and AR− whiteflies were determined using simple linear model with log-transformed data.

### Localisation of symbionts in nymphs and adults using FISH

The S-symbionts *Arsenophonus* and *Rickettsia* were localised in the cassava whitefly nymphs and adults by fluorescent in situ hybridisation (FISH) using specific probes labelled with cy3 or cy5 at the Volcani centre, Israel (Skaljac et al. 2010). AR+ whiteflies were used to localise *Arsenophonus* and *Rickettsia* and AR− as negative control. The MED population from Israel with triple infections with *Arsenophonus*, *Rickettsia* and *Portiera* were used as a positive control. The samples were visualised under a confocal microscope (IX81 Olympus FluoView 500 confocal microscope, Olympus Optical Co, Tokyo, Japan). Twenty-five nymphs and adults from each isofemale lines were tested for the location of symbionts in whitefly bodies.

## Results

### Fecundity and nymph development

**Fig. 1:**
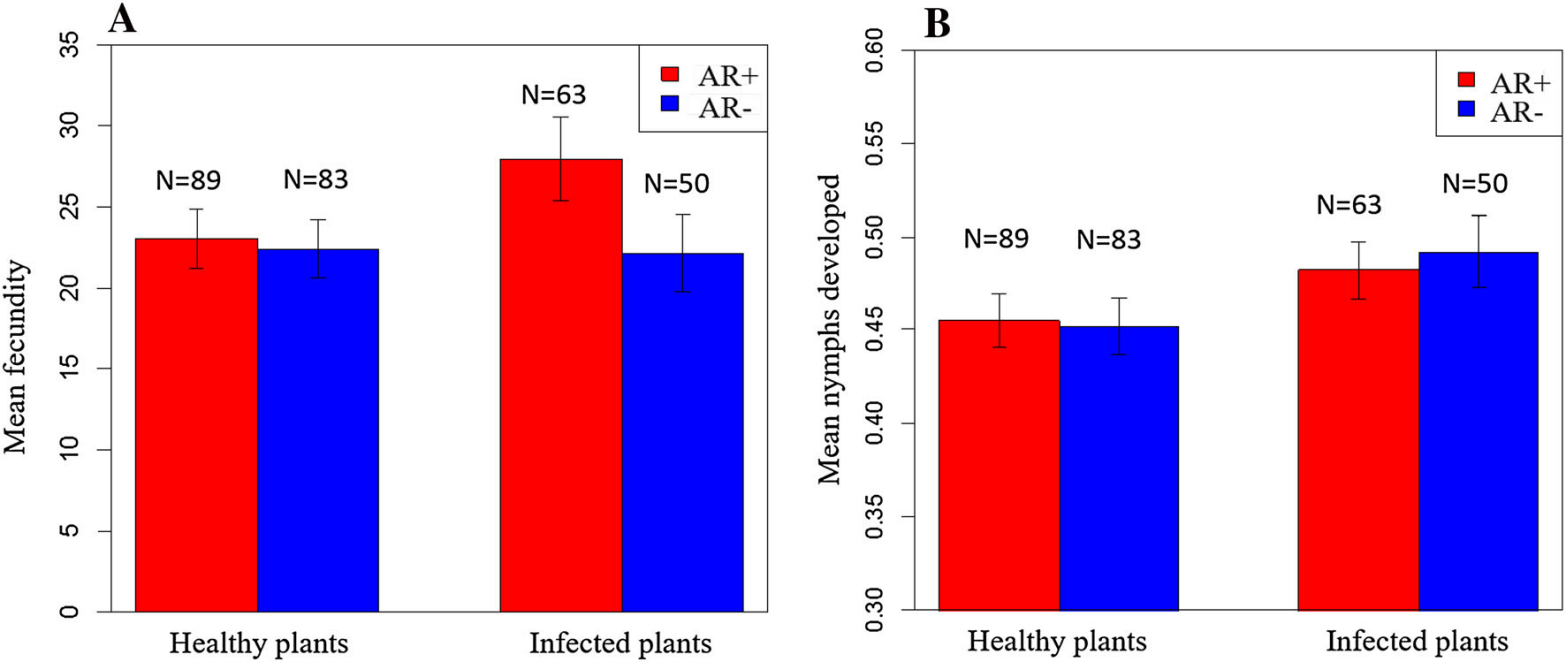
Mean fecundity (**a**) and proportion of nymphs developed (**b**) by SSA1-SG3 AR*+* and AR− whiteflies on healthy and EACMV-UG-infected cassava plants. No significant differences observed in fecundity and nymph development between AR*+* and AR− whiteflies

Infection of whiteflies by the two S-endosymbionts, or infection of cassava plants by EACMV-UG, had no significant effect on the fecundity of cassava whiteflies. However, AR+ whiteflies had slightly higher mean oviposition (27.9 ± 2.63 eggs per female) on virus-infected plants compared to healthy plants (23.0 ± 1.84 eggs). AR− whiteflies laid almost identical number of eggs both on virus-infected (22.4 ± 1.84 eggs) and healthy plants (22.1 ± 2.3 eggs; Fig. 1a) and thus were not statistically significant (Table S3 in Supporting Information). The mean proportion of nymphs developed by AR+ and AR− whiteflies was also similar on healthy plants, but slightly higher (*P* = 0.04) on virus-infected plants (Fig. 1b, Table S4 in Supporting Information).

### Adult emergence and development time

**Fig. 2:**
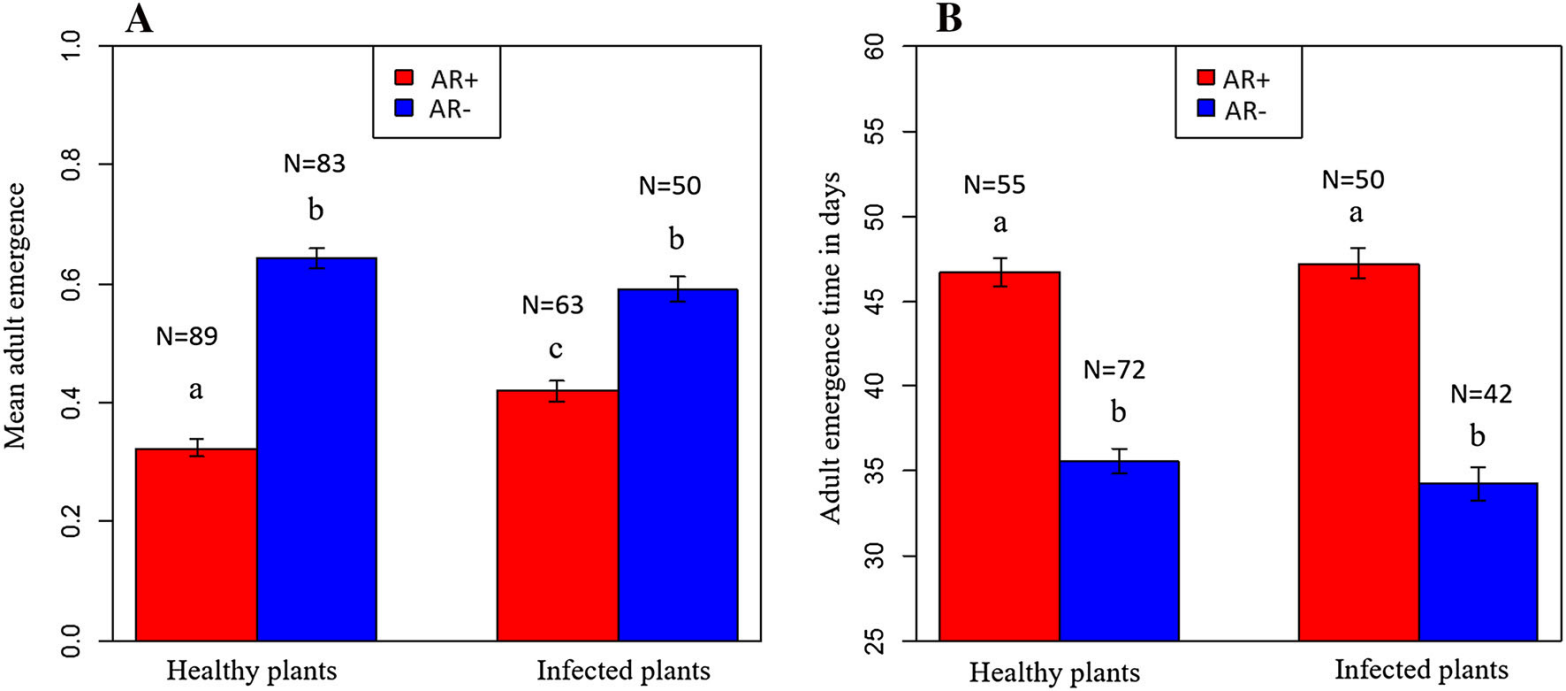
Mean proportion of adult emergence (**a**) and adult development time (**b**) by the SSA1-SG3 AR+ and AR− whiteflies on healthy and EACMV-UG infected cassava plants. Adult emergence was higher and quicker by AR− than AR+ flies

Mean proportion of adults emerged from AR− whiteflies on both healthy (0.64 ± 0.02) and virus-infected plants (0.59 ± 0.02) was significantly higher (*P* < 0.001) than AR+ on healthy (0.32 ± 0.01) and virus-infected (0.42 ± 0.02) plants (Fig. 2a, Table S5 in Supporting Information).

Mean adult development time was significantly shorter (*P* < 0.001) for AR− whiteflies on both healthy (35.5 ± 0.7 days) and virus-infected (34.2 ± 0.9 days) plants compared to AR+ whiteflies (46.7 ± 0.8 and 47.2 ± 0.9 days, respectively; Fig. 2b, see Table S6 in Supporting Information).

### Detection and quantification of EACMV-UG in single cassava whiteflies

**Fig. 3:**
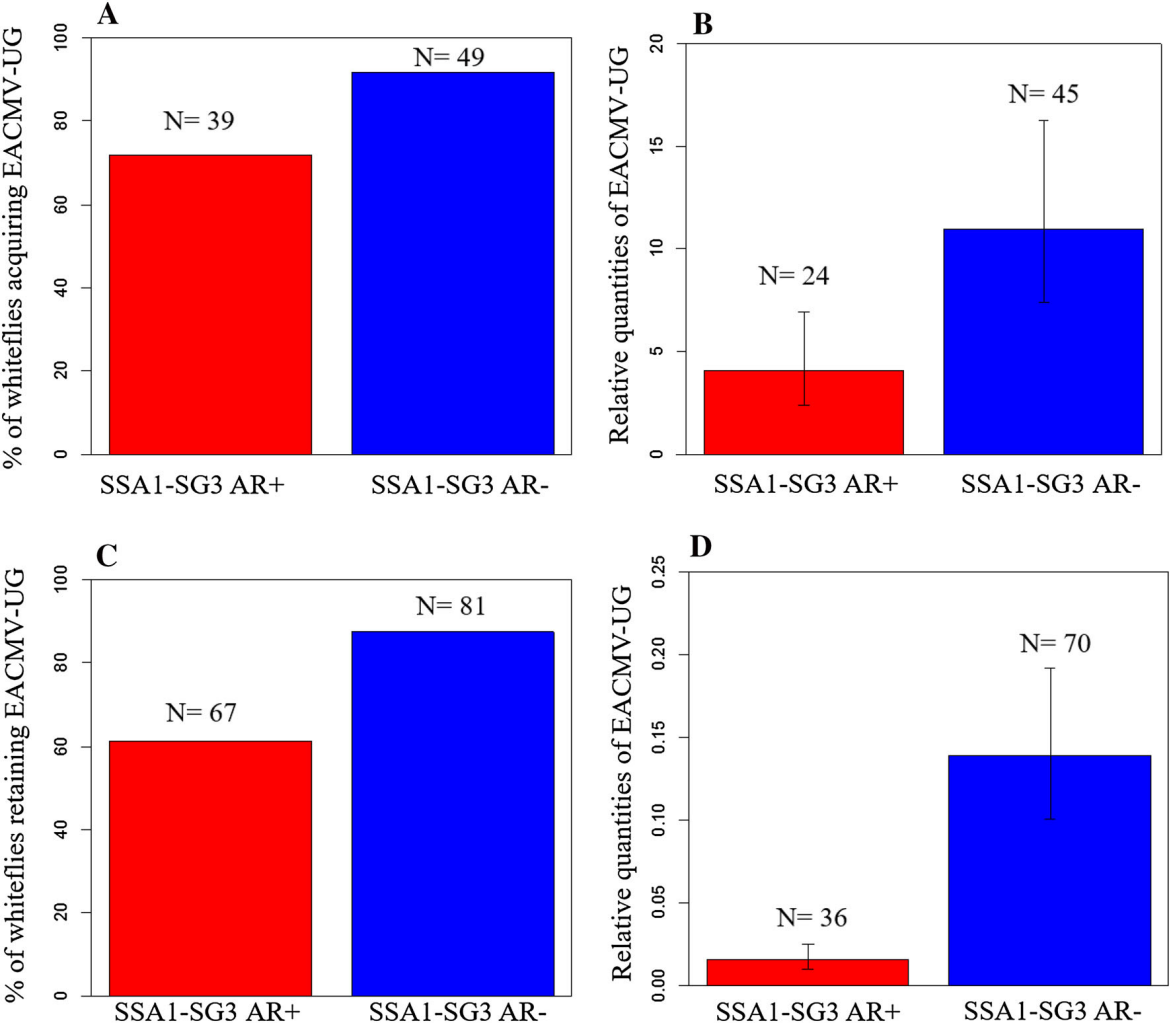
Percentage of SSA1-SG3 AR*+* and AR− whiteflies acquiring (**a**) and retaining (**c**) EACMV-UG after 48-h AAP and IAP, respectively. Relative quantities of EACMV-UG in AR*+* and AR− after 48-h AAP (**b**) and IAP (**d**), respectively. Higher proportion of AR− acquired and retained EACMV-UG than AR*+*. Quantities of virus acquired and retained was also higher in AR−

About 91.8% (45/49) of SSA1-SG3 AR− whiteflies acquired the virus after 48 h AAP, compared to 71.8% (28/39) of AR+ flies (*P* = 0.02; Fig. 3a). The mean relative quantities of virus acquired by AR− was also higher than AR+ whiteflies (Fig. 3b, see Table S7 in Supporting Information), but was statistically not significant (*F* = 2.23, *P* = 0.14).

The percentage of whiteflies retaining EACMV-UG after 48-h IAP was similarly higher (*P* = 0.0002) for AR− (87.65%, 71/81) than AR+ (61.2%, 41/67) whiteflies (Fig. 3c). AR− also retained higher quantities (~ninefold) of virus than AR+ whiteflies (*F* = 14.59, *P* = 0.0002; Fig. 3d, see Table S7 in Supporting Information).

SSA1-SG3 AR− (37.1%, 13/35 plants) were more efficient in transmitting EACMV-UG to healthy cassava plants than AR+ whiteflies (17.2%, 5/29). The differences in transmission were, however, not statistically significant (*P* = 0.09) due to inconsistent transmission rates across replications.

### Relative expression of immune genes

**Fig. 4:**
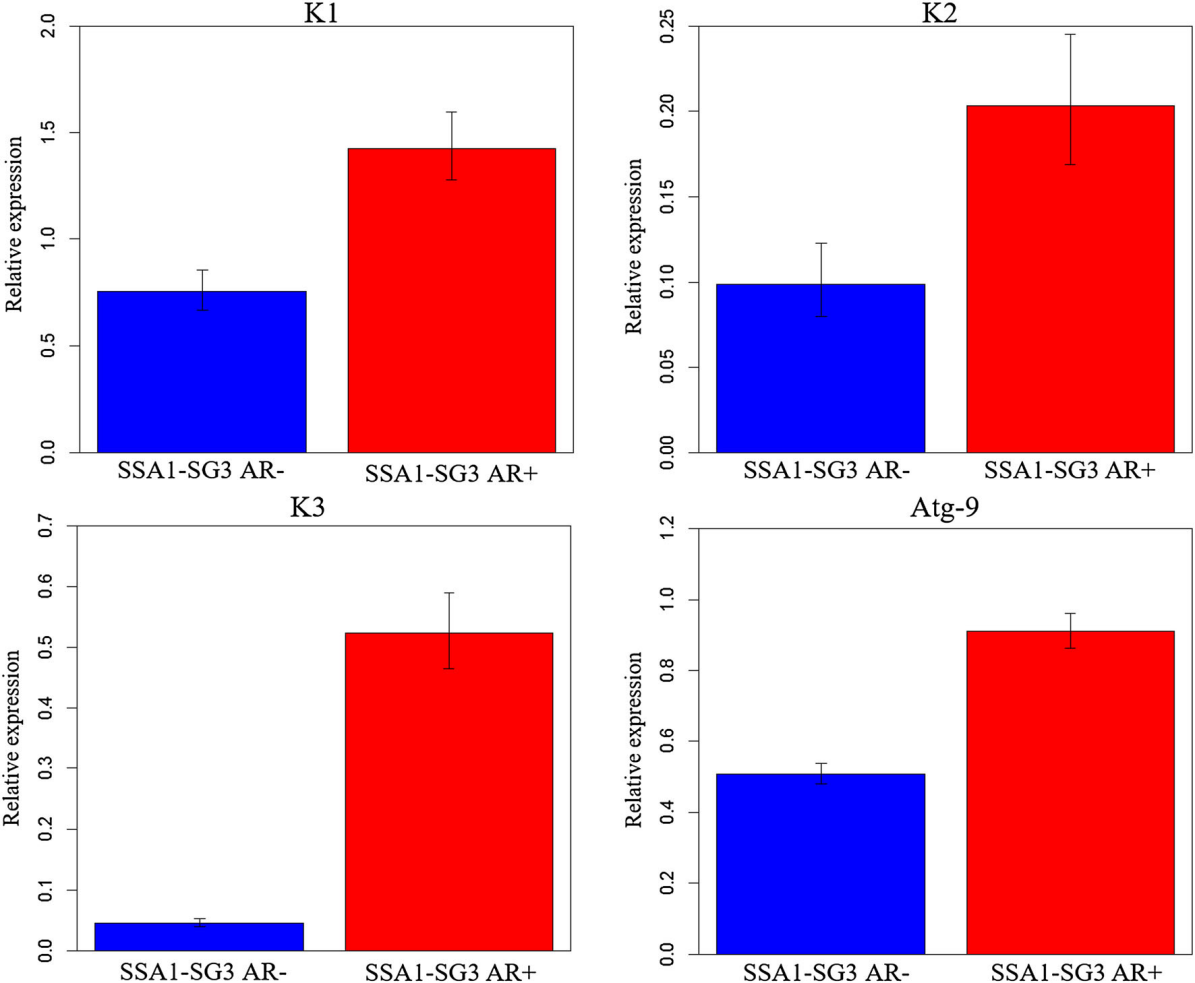
Relative expression of immune genes in SSA1-SG3 AR*+* and AR− whiteflies. Expression of antimicrobial peptides and autophagy protein was higher in SSA1-SG3 AR*+* than AR−. AR*+* infections induced higher immune response in SSA1-SG3 whiteflies

All the three antimicrobial peptides Knottin1 (*F* = 14.52, *P* ≤ 0.001), Knottin2 (*F* = 6.47, *P* = 0.015) and Knottin3 (*F* = 184, *P* ≤ 0.001) were overexpressed by minimum of twofold in AR+ compared to AR− populations (Fig. 4). The autophagy-related gene (*atg*-*9*) was also upregulated in AR+ populations (*F* = 184, *P* ≤ 0.001; Fig. 4).

### Localisation of symbionts in cassava whitefly nymphs and adults

**Fig. 5:**
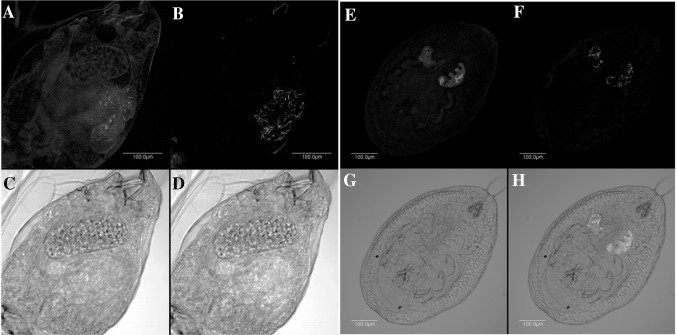
Localisation of *Rickettsia* and *Arsenophonus* in adults and nymphs of SSA1-SG3 AR*+*. *Rickettsia* (*red*) in adult (**a**) and nymph (**e**) in *dark field*; *Arsenophonus* (*blue*) in adult (**b**) and nymph (**f**) in *dark field*; adult (**c**) and nymph (**g**) in *bright field*; overlay of *Rickettsia* and *Arsenophonus* in adult (**d**) and nymph (**h**) in *bright field*. (Color figure online)

**Fig. 6:**
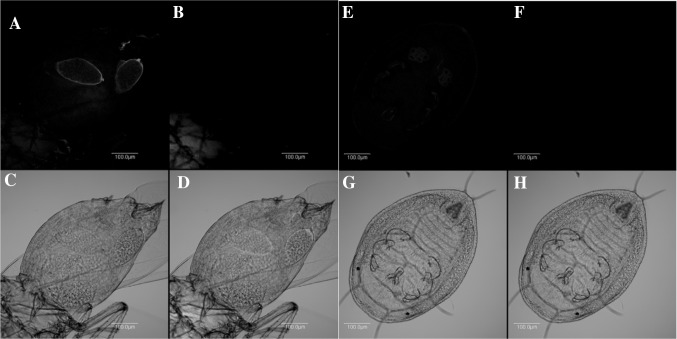
Localisation of *Rickettsia* and *Arsenophonus* in adults and nymphs of SSA1-SG3 AR−. *Rickettsia* (*red*) undetected in adult (**a**) and nymph (**e**) in *dark field*; *Arsenophonus* (*blue*) undetected in adult (**b**) and nymph (**f**) in *dark field*; adult (**c**) and nymph (**g**) in *bright field*; overlay of *Rickettsia* and *Arsenophonus* in adult (**d**) and nymph (**h**) in *bright field*. (Color figure online)

*Arsenophonus* and *Rickettsia* were detected in SSA1-SG3 AR+ nymphs and adults (Fig. 5), both confined inside bacteriocytes. *Portiera*, the P-symbiont, was detected in all samples (data not shown). *Arsenophonus* and *Rickettsia* could not be detected in SSA1-SG3 AR− whiteflies (Fig. 6), but *Portiera,* the P-symbiont, was detected in all samples (data not shown).

## Discussion

The phenotype of cassava whiteflies (SSA1-SG3) harbouring both *Arsenophonus* and *Rickettsia* was investigated to understand their implications on host biology and virus transmission abilities. In our conditions, AR+ infections had no effect on whitefly fecundity or nymph development. However, they had significant negative effect on adult development and generation time. AR+ infections caused 50% reduction in the number of nymphs developing into adults, and the adult development time was delayed by 10 days compared to AR− whiteflies. Both these parameters, if operational in cassava fields, will have significant negative impact on the whitefly population development and thus its pest status. The SSA1-SG3 used in this study has been found only in coastal areas of eastern Africa (Mugerwa et al. 2012; Legg et al. 2013; Tajebe et al. 2015a), a region where whiteflies on cassava are less abundant. A similar 50% reduction in *Drosophila* and mosquitoes population was seen when infected with *Wolbachia* (Min and Benzer 1997; McMeniman and O’Neill 2010). High numbers (84%) of SSA1-SG3 harboured *Arsenophonus* and/or *Rickettsia* infections in cassava fields (Ghosh et al. 2015). It is thus possible that the negative effects of these bacteria have kept these populations under control in the cassava-growing areas of coastal eastern Africa.

Another major biotic factor in the cassava pathosystem is the presence of several species of viruses. Cassava mosaic begomoviruses that cause CMD are widespread throughout the cassava-growing regions of Africa. Infection of cassava plants by the begomovirus EACMV-UG provide nutritional benefits (increases amino acid contents in infected leaves) to *B. tabaci* and thus enhance their population development (Colvin et al. 2006). Development of whiteflies on cassava is thus the result of several interacting factors including viruses and host-associated bacteria. We therefore investigated the interaction between whitefly associated endosymbiotic bacteria and EACMV-UG infections of cassava plants on the cassava whitefly biology. Our results indicated that infections of cassava plants by the EACMV-UG provided only marginal benefits to cassava whitefly development and slightly enhanced (3–4%) nymph emergence from eggs. The observed differences between this study and Colvin et al. (2006) are difficult to explain because of the fundamental differences in the two studies. Colvin et al. (2006) used an heterogenous whitefly population collected from farmer’s cassava fields in Uganda whose identity was not known but was most likely to have contained SSA1-SG1 species based on the recent literature (Ghosh et al. 2015). In contrast, we used an isofemale line of SSA1-SG3 from the coastal Tanzania, which has not been described from Uganda. The SSA1-SG1 and SSA1-SG3 have exhibited different biological properties in the field, with the former being highly fecund than the latter. Another major difference was in the nature of the plants used in the experiments. In the current study, we used diseased plants obtained from infected cuttings and thus had pronounced symptoms by the time experiments were started. In Colvin et al. (2006), diseased plants were generated by whitefly inoculations and the fecundity of the whiteflies was studied as the disease was developing in the plant. This was considered to have significant effect on whitefly fecundity as the greatest biochemical changes (availability of free amino acids) in plants occur during the process of virus infection (Colvin et al. 2006). This could also be a contributing factor for the differences between the two studies.

Further investigations on virus acquisition and retention showed that a higher proportion of SSA1-SG3 AR− flies (91.8%) acquired and retained the virus than AR+ (71.8%). The AR− also retained higher quantities of virus. These results are also similar to those seen with mosquitoes in which infection by *Wolbachia* caused significant reductions in the acquisition and transmission of mosquito-transmitted dengue virus (Osborne et al. 2009). This has led to using certain strains of *Wolbachia* as biocontrol agents to reduce dengue transmission in Australia, Indonesia, Vietnam, Columbia and Brazil (http://www.eliminatedengue.com/project). Our results thus support the hypothesis of using bacteria as potential biocontrol agents to reduce the whitefly population development as well as the spread of CMD and CBSD in Africa.

To investigate the effect of bacterial infections on whitefly innate immune response, the expression of four genes was studied by qPCR analysis. Antimicrobial peptides (AMPs) such as Knottins are expressed constitutively in *B. tabaci* (Shatters et al. 2008) and are overexpressed after acquisition of begomoviruses and other pathogenic infections (Mahadav et al. 2008, 2009; Luan et al. 2011; Zhang et al. 2014; Shalev et al. 2016). Autophagy-related protein (Atg-9) is a major innate immune response in *B. tabaci* (Luan et al. 2011) and other insects such as *Drosophila melanogaster* against pathogenic infection from bacteria and virus (Yano et al. 2008; Shelly et al. 2009). The AR+ infections in cassava whiteflies triggered similar pathogenic response in the cassava whiteflies through the over expression of Knottins and Atg-9. These innate immune responses in insects are maintained at metabolic and physiological costs (Freitak et al. 2003, 2007; Ardia et al. 2012). Evolution of higher levels of immune defence was proposed to compromise fitness traits due to additional energy demands (Schmid-Hempel 2005). Thus, the compromise in fitness traits in AR+ could be a cost for maintenance of higher immune responses for regulation of symbionts. Although AR+ infections in this study had negative impact on its host, both *Arsenophonus* and *Rickettsia* had high prevalence in SSA1-SG3 (Ghosh et al. 2015). It is possible that symbiont infections provide other kinds of benefits to cassava whiteflies which were not measured in this study. One study has already shown that infection with *Rickettsia* in *B. tabaci* provides higher tolerance to heat, and this was positively correlated with the expression of cytoskeleton genes (Brumin et al. 2011). That study hypothesised that infection with *Rickettsia* possesses a stress on the whitefly leading to the expression of heat stress- and heat tolerance-related genes, thus indirectly making the whitefly tolerant to heat. Whether symbiont infections of cassava whitefly provide defence against biotic and abiotic stress warrant further investigation in future.

Fewer SSA1-SG3 AR+ flies acquired and retained EACMV-UG than SSA1-SG3 AR− populations. EACMV-UG detected after 48-h IAP is expected to have crossed the gut epithelium and thus circulate in the haemocoel of the insects (Ghanim et al. 2001). Passage and retention of higher titres of virus in the haemocoel is of great importance as they can solely be transmitted by the vector (Storey 1938) and emphasises the importance of symbionts in the epidemiology of the disease. The reason for the lower retention of virus in AR+ whiteflies is unknown although autophagy/lysosome proteins and antimicrobial peptides have been previously shown to be important for the degradation of begomovirus both in the whitefly and plant host (Luan et al. 2011; Miozzi et al. 2014; Gorovits et al. 2014). Silencing Atg-9 and Knottin1 gene in *B. tabaci* increased begomovirus load and transmission efficiency indicating its role in the regulating virus quantities inside the whitefly body (Shalev et al. 2016; Wang et al. 2016). Overexpression of Atg-9 and Knottins in SSA1-SG3 AR+ whiteflies thus could possibly result in greater degradation of EACMV-UG and could be the main cause of low virus retention. Better fitness traits of SSA1-SG3 AR− whiteflies and generally being healthier could also be the reason for its better virus retention and acquisition abilities. In contrast to the above results, *Rickettsia* in MEAM1 and *Hamiltonella* in MED species facilitate begomovirus transmission (Kliot et al. 2014) by protecting virions against the hostile proteolytic haemolymph environment while transit from the gut wall to the salivary glands (Ohnesorge and Bejarano 2009; Gottlieb et al. 2010). These results indicate the complexity of whitefly–endosymbiont–virus interactions and the importance of studying them to better understand cassava disease pandemics.

However, the transmission efficiency to healthy plants in this study was very low and no significant difference was seen between AR+ and AR− whiteflies. Infection of the plants by EACMV-UG was confirmed by qPCR tests, although the Cq values of virus in plants was above 35 cycles, which is in the borderline of detection limit. This suggests that the virus was transmitted by whiteflies but was not multiplying in plants. Insect transmissibility of whitefly transmitted begomoviruses can be rapidly lost by prolonged vegetative propagation in absence of the vector (Liu et al. 1997). EACMV-UG virus used for inoculation in this study was maintained by repeated vegetative propagation for over 15 years in the absence of vector, and this possibly accounts for the low insect transmission and multiplication of virus inside plants.

*Arsenophonus*, *Rickettsia* as well as *Portiera* were detected in the bacteriocytes of cassava whitefly nymphs and adults. They were not scattered throughout the body as was seen in some other species (Gottlieb et al. 2006; Bing et al. 2014; Marubayashi et al. 2014). Coinfection of primary and secondary symbionts inside a common host-derived cell specially meant to accommodate symbionts offers several advantages to the tenants (Gottlieb et al. 2008). Escape of symbionts from the bacteriome to the body cavity triggers wide immune reactions in the host (Anselme et al. 2008; Reynolds and Rolff 2008), while expression of immune genes inside the bacteriome is minimal (Anselme et al. 2008). Thus, sharing of space inside the bacteriome could be an adaptation by the symbionts to evade the whitefly immune system. However, sharing common space has its downfall. This creates competition for space and nutrition (Vautrin and Vavre 2009), resulting in lower density of P-symbiont which can be detrimental to host fitness (Gottlieb et al. 2008). This could also be a possible reason for the negative impact of AR+ infections on cassava whiteflies. Our previous study on the prevalence of symbionts (Ghosh et al. 2015) shows that single infections of S-symbionts (59% of total infections) were more prevalent than double (37%) and triple infections (4%) in cassava whiteflies. This could be due to competition for space inside the bacteriocyte, whereas in invasive MEAM1 and MED populations where scattered phenotypes of symbionts were also found (Gottlieb et al. 2006; Skaljac et al. 2010; Marubayashi et al. 2014), the frequency of multiple infections were high (Chiel et al. 2007; Gueguen et al. 2010).

In conclusion, our results provide the additional evidence of the three-way interactions between the whitefly host, endosymbionts and plant viruses. Absence of S-endosymbionts in the cassava whitefly had positive effects on their fitness and vector abilities. High numbers of the currently superabundant populations are also free of S-endosymbionts, thus indicating the possible role of endosymbionts on keeping a check on cassava whitefly populations. This gives us the opportunity to use bacterial endosymbionts as potential biocontrol agents on cassava whiteflies and virus diseases.

## Author contributions

SG generated whitefly isofemale lines, performed research, analysed data and wrote the first draft of the manuscript. SB helped with design, analysis and revision of manuscript. SR and MG helped with the fluorescent microscopy. MNM conceived the work, collected samples, generated whitefly isofemale lines, designed research and corrected the manuscript extensively.

## Electronic supplementary material

Below is the link to the electronic supplementary material.

Supplementary material 1 (DOCX 20 kb)
