# Supplementary material for: Fitness costs associated with infections of secondary endosymbionts in the cassava whitefly species Bemisia tabaci
Source: J Pest Sci (2004). 2017 Aug 22;91(1):17–28. doi: 10.1007/s10340-017-0910-8 (PMC5750334; doi:10.1007/s10340-017-0910-8)
Supplement: Supplementary file 1 — Supplementary material 1 (DOCX 20 kb) [file 10340_2017_910_MOESM1_ESM.docx]

Table S1: Primers used for quantifying EACMV-UG in cassava whitefly.

| Target | Primer/ probe sequence (5’→3’) | Product | Reference |
| --- | --- | --- | --- |
| α- Tubulin  *B. tabaci* | F- TGTACCGAGGAGATGTTGTG  R- GATACCGACCTTGAAACCAG  Probe- Cy5- ATGCTGCCATCGCCACCATC- BHQ3 | 113 bp | This study |
| EACMV/ EACMV-UG | CMBRep/F- CRTCAATGACGTTGTACCA  Neweac-alt/R- CATGGAGACCGATCAGTATTGTTC  Probe- FAM- TCTTKGGAG/  ZEN/ACAGATCCAGGTGTCCACAT-IABkFQ | 113 bp | Otti *et al*., 2016 |

Table S2: Primers used for quantifying whitefly immune genes.

| **Target gene** | **Primer sequence (5’→3’)** | **Product size** | **Function** | **Reference** |
| --- | --- | --- | --- | --- |
| α- Tubulin  *B. tabaci* | F- TGTACCGAGGAGATGTTGTG  R- GATACCGACCTTGAAACCAG | 113 bp | Structural protein | This study |
| Knottin 1 (K1) | F- TCCTGGACATTGCACAACCA  R- TTTCGGAGGGATTGGAATGA | 81 bp | Antimicrobial peptide | Mahadav *et al*., 2009 |
| Knottin 2 (K2) | F-CTGTTCCAAGCCAAAACCGA  R-GATCATGAAGGCGGCCACTA | 81 bp | Antimicrobial peptide | Mahadav *et al*., 2008 |
| Knottin 3 (K3) | F- CATGGTCGCTGTCAACGTCT  R- TTGCAACTGGCACCTTTGG | 81 bp | Antimicrobial peptide | Mahadav *et al*., 2009 |
| *atg-9* | F- AGGGTTCCTGGTTCACGC  R-TTGCCATCATTAACTTTCTGCT |  | Autophagy | Luan *et al*., 2011 |

Table S3: Analysis of deviance on the fecundity of AR+ and AR- whiteflies on healthy and EACMV-UG infected cassava plants

|  | df | Deviance | Residual df | Residual deviance | *P* value (Chi) |
| --- | --- | --- | --- | --- | --- |
| symbiont | 1 | 1.70 | 284 | 305.88 | 0.19 |
| virus | 1 | 1.21 | 283 | 304.66 | 0.27 |
| symbiont:virus | 1 | 1.28 | 282 | 303.38 | 0.25 |

Table S4: ANOVA on mean proportions of nymphs developed for AR+ and AR- whiteflies on healthy and EACMV-UG infected cassava plants.

|  | df | Deviance | Residual df | Residual deviance | *F* value | *P* value |
| --- | --- | --- | --- | --- | --- | --- |
| symbiont | 1 | 0.003 | 284 | 562.28 | 0.002 | 0.96 |
| virus | 1 | 7.02 | 283 | 555.26 | 4.17 | 0.04* |
| symbiont:virus | 1 | 0.25 | 282 | 555.00 | 0.15 | 0.69 |

Table S5: Multiple comparison of mean proportion of adult emergence (Tukey’s HSD test).

| Comparison | | Standard error | *Z* value | *P* value |
| --- | --- | --- | --- | --- |
| AR- on healthy plants | AR+ on healthy plants | 0.1 | 13.08 | < 0.001** |
| AR+ on virus plants | AR+ on healthy plants | 0.09 | 4.18 | < 0.001** |
| AR- on virus plants | AR+ on healthy plants | 0.1 | 9.87 | < 0.001** |
| AR+ on virus plants | AR- on healthy plants | 0.1 | -9.05 | < 0.001** |
| AR- on healthy plants | AR- on virus plants | 0.1 | -1.93 | 0.2 |
| AR- on virus plants | AR+ on virus plants | 0.1 | 6.2 | < 0.001** |

Table S6: Multiple comparison of mean duration of adult emergence (Tukey’s HSD test).

| Comparison | | *P* value |
| --- | --- | --- |
| AR- on healthy plants | AR+ on healthy plants | < 0.001** |
| AR+ on virus plants | AR+ on healthy plants | < 0.98 |
| AR- on virus plants | AR+ on healthy plants | < 0.001** |
| AR+ on virus plants | AR- on healthy plants | < 0.001** |
| AR- on healthy plants | AR- on virus plants | 0.7 |
| AR- on virus plants | AR+ on healthy plants | < 0.001** |

Table S7: Mean relative quantities of EACMV-UG in single whiteflies after 48 hours AAP and IAP in SSA1-SG3 AR+ and AR-

|  | Mean ± SE of EACMV-UG quantity | |
| --- | --- | --- |
|  | AAP | IAP |
| SSA1-SG3 AR- | 10.97 ± 4.43 | 0.13 ± 0.045 |
| SSA1-SG3 AR+ | 4.04 ± 2.29 | 0.015 ± 0.007 |
